# Multidisciplinary management of clival chordomas; long-term clinical outcome in a single-institution consecutive series

**DOI:** 10.1007/s00701-017-3266-1

**Published:** 2017-07-22

**Authors:** Petter Förander, Jiri Bartek, Michael Fagerlund, Hamza Benmaklouf, Ernest Dodoo, Alia Shamikh, Pär Stjärne, Tiit Mathiesen

**Affiliations:** 1 Department of Neurosurgery, Karolinska University Hospital, Stockholm, Sweden; 2 Department of Clinical Neuroscience, Karolinska Institutet, Stockholm, Sweden; 3 Department of Neurosurgery, Copenhagen University Hospital Rigshospitalet, Copenhagen, Denmark; 4 Department of Neuroradiology, Karolinska University Hospital, Stockholm, Sweden; 5 Department of Physics, Karolinska University Hospital, Stockholm, Sweden; 6 Department of Pathology, Karolinska University Hospital, Stockholm, Sweden; 7 Department of Otorhinolaryngology, Karolinska University Hospital, Stockholm, Sweden

**Keywords:** Chordoma, Microsurgery, Endonasal, Transsphenoidal, Gamma Knife radiosurgery, Skull base

## Abstract

**Objective:**

Chordomas of the skull base have high recurrence rates even after radical resection and adjuvant radiotherapy. We evaluate the long-term clinical outcome using multidisciplinary management in the treatment of clival chordomas.

**Methods:**

Between 1984 and 2015, 22 patients diagnosed with an intracranial chordoma were treated at the Karolinska University Hospital, Stockholm, Sweden. Sixteen of 22 were treated with Gamma Knife radiosurgery (GKRS) for tumour residual or progression during the disease course. Seven of 22 received adjuvant fractionated radiotherapy and 5 of these also received proton beam radiotherapy.

**Results:**

Fifteen of 22 (68%) patients were alive at follow-up after a median of 80 months (range 22–370 months) from the time of diagnosis. Six were considered disease free after >10-year follow-up. The median tumour volume at the time of GKRS was 4.7 cm^3^, range 0.8–24.3 cm^3^. Median prescription dose was 16 Gy, range 12–20 Gy to the 40–50% isodose curve. Five patients received a second treatment with GKRS while one received three treatments. After GKRS patients were followed with serial imaging for a median of 34 months (range 6–180 months). Four of 16 patients treated with GKRS were in need of a salvage microsurgical procedure compared to 5/7 treated with conventional or proton therapy.

**Conclusion:**

After surgery, 7/22 patients received conventional and/or photon therapy, while 15/22 were treated with GKRS for tumour residual or followed with serial imaging with GKRS as needed upon tumour progression. With this multidisciplinary management, 5- and 10-year survivals of 82% and 50% were achieved, respectively.

## Introduction

Intracranial chordomas are rare tumours arising extraduraly in the clivus and midline structures of the skull base and the yearly incidence is no more than 0.1/1000.000 [35]. Histopathological features of the tumours indicate remnants of the notochord as the origin [46]. Recent gene expression studies analysing cytokeratins and brachyury proteins further strengthen this theory [52, 60]. Although considered low-grade malignancies, these tumours are locally aggressive with invasive growth within mesenchymal-derived tissues and a high risk of eventual demise.

Microsurgery, with the aim of gross total (GTR) or subtotal (STR) resection, followed by radiotherapy is considered the standard treatment for clivus chordomas [19, 22, 42, 49, 51]. Most chordoma patients are treated with transsphenoidal resection [14, 16, 23, 28, 50, 54], although more extensive tumours require transbasal or transpetrosal approaches [1]. Since only retrospective case series are available in literature, the warrants for microsurgery and adjuvant radiotherapy are weak.

It is well accepted that these centrally located tumours in the skull base are often impossible to radically remove without high risk of causing neurological deficits [18, 44, 56]. Chordomas are usually surrounded by invading cells in surrounding bone and recurrences are frequent even after presumed radical surgery. Still, our current knowledge of chordomas forms a hermeneutic rationale for a combination of microsurgery and adjuvant radiation. Chordomas respond poorly to conventional radiotherapy [36, 62], and therefore linear accelerator (LINAC)-based fractionated stereotactic radiotherapy (FSRT) [10] intensity-modulated radiation therapy (IMRT) [45] and proton-beam radiotherapy [2–4, 20, 21, 37, 39, 40] have been used. Gamma Knife radiosurgery (GKRS) has a potential to control tumours with poor radiation sensitivity and has, accordingly, also been reported to offer an effect on chordomas [19, 22, 26, 27, 33, 38, 53].

Due to the rarity of chordomas, even large centres deal with a small number of patients each year. Neurosurgical and oncological management has evolved gradually [2], driven by local resources and facilities. Major management differences are possible in spite of widespread consensus regarding surgery, the natural history and the need of adjuvant non-surgical treatment. Large-scale prospective studies may be of benefit, but in the past and in the foreseeable future, scientific warrant for chordoma management must rely on critical analyses of available retrospective data.

Our understanding of the present consensus is that most specialists agree on three points: (1) “safe” microsurgery is indicated for all patients, although small, histopathologically verified tumours can be treated with only stereotactic radiosurgery (SRS) [19, 26, 27, 53], (2) despite extensive surgical removal and adjuvant treatment, tumour recurrence/progression is common, and (3) chordomas require adjuvant treatment following initial therapy, often in close proximity to the index surgery [5].

Three issues are controversial: (1) Should radiation therapy be initiated in close proximity to index surgery, or at a later stage? (2) Is there a difference between response to photons versus heavy particles, or are the total dose, fractionation and dosimetric distribution of main importance? (3) Should SRS be used as a complement to microsurgery or as salvage therapy in case of progressive tumour growth?

The resources and healthcare organisation of our catchment area have provided an infrastructure that has led to management that differs somewhat from the consensus outlined above, especially regarding early radiotherapy. In addition, the invention and early development of GKRS [30, 31] at our centre have provided easy access for patients with radiosurgically accessible lesions, such as clival chordomas. We report the long-term results of microsurgery and adjuvant treatments, with emphasis on close follow-up and the use of GKRS, for patients with intracranial chordomas treated consecutively between 1984 and 2015 at Karolinska University Hospital, Stockholm, Sweden. The main aim is to determine whether this management provides similar or different long-term outcomes than management strategies that include early heavy-particle radiation following gross total removal.

## Methods

### Demographic characteristics

From 1984–2015, 27 consecutive patients with a preliminary histopathological diagnosis of intracranial chordomas were treated at the Karolinska University Hospital, Stockholm, Sweden. Of these, five patients were excluded from further analyses; three patients were referred from abroad for complementary GKRS after initial microsurgical treatment in their country of origin and were subsequently lost to follow-up, while two patients were excluded when the review of pathological diagnosis revealed that their tumours were in fact chondrosarcomas (Table 1). The remaining 22 patients were all treated with microsurgery as first-line treatment at the Karolinska University Hospital (*n* = 15) or at one of the referring neurosurgical centres in Sweden (*n* = 7), allowing for a definitely confirmed histopathological diagnosis following re-examination, renewed immunohistochemistry and diagnosis confirmation.

**Table 1:** Demographic, treatment and outcome data

Patient no.	Age/sex	Location	Volume (cm^3)^/size*	Symptoms**	KPS	No. of surgical procedures	No. of fractionated treatments	Resection grade	Permanent neurological deficit	Diagnosis and PI	No. of GKRS treatments	No. of proton beam RT treatments	Surgical approaches	Time to GKRS, fractionated treatment, proton beam RT or reoperation after index surgery	Outcome/clinical follow-up
1	62/M	Cl, CP	14.0 Small	Cn.IV, cn.VI	90	4	1	Partial	0	Chordoma, PI < 7%	1	1	Trsf × 3, Pter ×1	4 months (reoperation) 6 months (proton) 57 months (GKRS)	Alive/ 98 months
2	16/M	Cl, MF, AP, CP,	63.0 Large	Headache, cn.VII, cn.VIII	90	8	1	Partial	0	Chordoma	2	1	LR ×2, Pter ×1, RS ×3, TP ×2	41 months (reoperation) 84 months (proton) 116 months (GKRS)	Dead/196 months
3	53/M	Cl	27.6 Large	Diplopia, cn.III, cn.V	90	1	1	STR	cn III	Chordoma	0	1	Transbasal midline ×1	7 months (proton)	Dead/ 36 months
4	64/F	Cl	4.0 Small	Diplopia, cn.VI	90	1	0	Partial	0	Chondroid chordoma	1	0	Trsf ×1	7 months (GKRS)	Alive/ 47 months
5	41/F	Cl, CS, MF	33.0 Large	Hemi- anopsia, cn.V, cn.VI	90	2	1	STR	0	Chordoma	1	0	Pter ×1	2 months (FRT) 62 months (reoperation) 72 months (GKRS)	Alive/ 370 months
6	59/M	Cl, PS, SP	1.6 Small	Epidural fistula	100	3	0	Partial	0	Chordoma, PI 25%	1	0	Trsf ×1 and biopsy ×2	22 months (GKRS)	Alive/70 months
7	33/F	Cl	75.0 Large	–	100	3	0	GTR	0	Chordoma	0	0	Trsf ×1, transbasal ×1	–	Alive/ 100 months
8	55/M	Cl	ND Small	–	100	4	0	STR	0	Chordoma	1	0		6 months (reoperation) 22 months (GKRS)	Dead/ 293 months
9	28/M	Cl	ND Large	Diplopia, cn.VI	100	1	0	STR	0	Chordoma	1	0	Trsf ×1	14 months (GKRS)	Alive/ 120 months
10	39/M	Cl, AP, CS	2.2 Small	Diplopia Retroorbital pain, cn.VI	90	2	0	STR	0	Chordoma	3	0	Trsf ×1	36 months (GKRS) 84 months (reoperation)	Alive/ 37 months
11	57/M	Cl	NA Small	Hemiparesis	90	1	0	GTR	0	Chordoma, PI 2%	0	0	Combined TP ×1	–	Alive/67 months
12	31/F	Cl, CS, NP	35.5 Large	Diplopia, cn.VI	90	4	0	STR			2	0	Pter × 2, bilat. LR ×1	11 months (reoperation) 22 months (GKRS)	Dead/ 83 months
13	14/F	Cl	25.4 Large	Pain, progression of hemi−/ tetraparesis	100	3	1	STR	0	Chordoma	1	0	Maxilotomi ×3, SO ×1	24 months (reoperation) 8 months (GKRS) 36 months (FRT)	Dead/ 77 months
14	79/F	Cl	0.9 Large	Diplopia, ptosis, cn.III	100	2	0	Partial	0	Chordoma	1	0	Trsf ×2	14 months (reoperation) 21 months (GKRS)	Dead/ 55 months
15	79/F	Cl	6.0 Small	Diplopia, cn.III	90	1	0	STR	0	Chordoma	1	0	Trsf ×1	13 months (GKRS)	Alive/ 105 months
16	56/F	Cl, CS	NA Large	Facial numbness, cn.V	100	5	1	STR	0	Chordoma, PI 2% with hot spots	0	1	TB ×2, Pter ×1,	6 months (proton) 35 months (reoperation)	Dead/ 72 months
17	40/M	Cl, SC, CP, MF	17.9 Large	Dysartria, Vertigo, cn.V	100	1	0	Partial	0	Chondroid chordoma	1	0	Pter ×1	8 months (GKRS)	Alive/ 27 months
18	31/M	Cl,	13,9 Large	Diplopia, cn.VI	90	4	0	STR	0	Chordoma	1	0	Trsf ×2, Pter ×1, Combined TP ×1	21 months (reoperation) 37 months (GKRS)	Alive/ 36 months
19	76/F	Cl, MF, AP	NA Large	Dysphagia, diplopia	70	1	0	STR	Cn IX, cn X	Chordoma	2	0	Translab. ×1	35 months (GKRS)	Alive/ 116 months
20	56/F	Cl, CS	3.6 Large	Visual deficit, cn.II	90	1	0	GTR	0	Chordoma, PI <10%	0	0	Trsf ×1	–	Alive/ 13 months
21	44/F	Cl, AP, CP	38,5 Large	Hemiparesis, Cn.VII	60	2	0	STR	0	Chordoma	2	0	Trsf ×1, Pter ×1	3 months (reoperation) 7 months (GKRS)	Alive/ 36 months
22	40/M	Cl, PCl	3,7 Small	Diplopia, cn.IV	100	1	1	Biopsy	0	Chondroid chordoma, PI 1%	0	1	Trsf ×1 and biopsy ×1	120 months (proton)	Alive/123 months

*Size small 3≤ cm vs. large >3 cm, **symptoms and cranial nerve palsy at diagnosis

*Abbreviations*: *Cl* clivus, *MF* middle fossa, *PS* parasellar, *SP* sphenoideal, *CP* cerebello pontine, *CS* cavernous sinus, *PCl* petro-clival, *NP* naso-pharyngeal, *NA* not available, *cn* cranial nerve, *STR* subtotal resection, *GTR* gross total resection, *KPS* Karnofsky performance status, *GKRS* Gamma Knife radiosurgery, *PI* proliferation index, *Trsf* transsphenoidal, *Pter* pterional, *LR* lateral rhinotomy, *RS* retrosigmoid, *TP* transpetrosal, *OZ* orbitozygomatic, *SO* suboccipital, *TB* transbasal

Maximal safe cyto-reductive microsurgery was the goal for the initial treatment, although biopsies were only performed in two patients. Surgical approaches were tailored according to the location and relation of the tumour to critical neurovascular structures. Altogether 55 microsurgical treatments were performed for the 22 patients (range 1–8 procedures per patient). The transsphenoidal approach was the most common approach (17 of 55), followed by the pterional/orbitozygomatic (10 of 55), combined infra- and supratentorial (5 of 55), transbasal (4 of 55) and suboccipital/retrosigmoidal (3 of 55). Complications after surgery were classified [29] according to Ibanez et al., where Ibanez grade I represents any non-life-threatening complication treated without invasive procedures, grade II is complications requiring invasive management, grade III is life-threatening adverse events requiring treatment in an intensive care unit (ICU) and grade IV is deaths as a result of complications.

### Gamma Knife radiosurgery

GKRS was performed in 16 out of 22 patients at a median time of 22 (range, 7–116) months after the first microsurgical treatment. No patients received GKRS prior to microsurgery. Since none of the microsurgery was considered radical, the indication for GKRS was tumour recurrence/progression after watchful waiting with serial imaging (*n* = 10) or treatment of the tumour residual (*n* = 6). Ten patients received one GKRS treatment; five patients two GKRS treatments, while one patient received three GKRS treatments. The GKRS procedure has been described previously [31, 57]. Briefly, a Leksell stereotactic frame (G-frame) and Leksell Gamma Knife®, models B and C (Elekta, Stockholm, Sweden) were used. In 2009, the latest version of the Gamma Knife, Perfection™, was installed and has been used since. The stereotactic frame was applied under local anaesthesia, following administration of oral benzodiazepine. Stereotactic MRI was used for dose planning in all patients, although the MRI sequences have changed over the years. Fourteen of the Gamma Knife procedures were performed with Leksell Gamma Plan®, (Elekta, Stockholm, Sweden), while five procedures between 1990 and 1993 were carried out with the Kula dose planning software system (Elekta, Stockholm, Sweden). The median target volume was 4.7 cm^3^ (range 0.8–24.3 cm^3^). The median prescription dose was 16 Gy (range 12–20 Gy). The median maximum dose to the tumour volume was 36 Gy (range 28–50 Gy). After the Gamma Knife treatment the stereotactic frame was removed and the patients were discharged after a few hours of observation.

Regarding radiation treatment: (1) GKRS was the most frequently implemented radiation modality, (2) five patients were treated with proton beam radiotherapy, which was delivered as a combination of fractionated combined proton and photon radiation therapy, and (3) two patients were treated with fractionated radiotherapy (FRT) at a total dose of 50–54 Gy in fractions of 1.8–2.0 Gy.

### Follow-up

The patients were followed clinically for a median of 80 months (range 22–370 months) after index microsurgery. Radiologically, patients were followed with MRI for a median of 77 months (range 12–311 months). The post-operative MRI was performed within 3 months after surgery and then annual MRI controls were performed for a period of 5 years, after which an individual follow-up plan was tailored. The result of microsurgery was classified as GTR (no visible residual), STR (visible residual approximately <5% of original tumour size on contrast-enhanced MRI), partial removal (visible residual, 5–50% of original tumour size) or biopsy (visible residual >50% of original tumour size). Any increase in tumour volume was considered a “tumour recurrence/progression” and patients were subsequently evaluated at a multidisciplinary conference for possible additional treatment. The tumor volume wasmeasured on LGP software or estimated by measuring the tumour radius (r) in three dimensions on MR sections (using the formula V = 4/3π × r1 × r2 × r3). Images that allowed exact measurements at diagnosis were unavailable for five patients. For this reason, we also classified tumours as small (≤3 cm in maximal diameter) or large (> 3 cm), since this information was available for all patients. In the 17 patients receiving GKRS, follow-up after the initial GKRS treatment ranged from 6 to 180 months (median of 34 months). Overall survival (OS) after diagnosis was the primary endpoint. Results are reported as cumulative survival plots with estimates of median and mean survival.

### Ethics

The study was approved by the regional ethical committee in Stockholm, Sweden (no. 2016/1497–31/4).

## Results

### Patients, surgical outcome and overall survival

At the time of diagnosis, the median age of the patients (*n* = 22) was 48 years (range 14–79 years), and 11 were male. The median Karnofsky Performance Status (KPS) score was 90 (range 60–100). Cranial nerve palsy was the most frequent objective finding, with 12/22 patients experiencing diplopia; 7 of these were caused by abducens nerve palsy, 3 by oculomotor nerve palsy and 2 by trochlear nerve palsy.

**Fig. 1:**
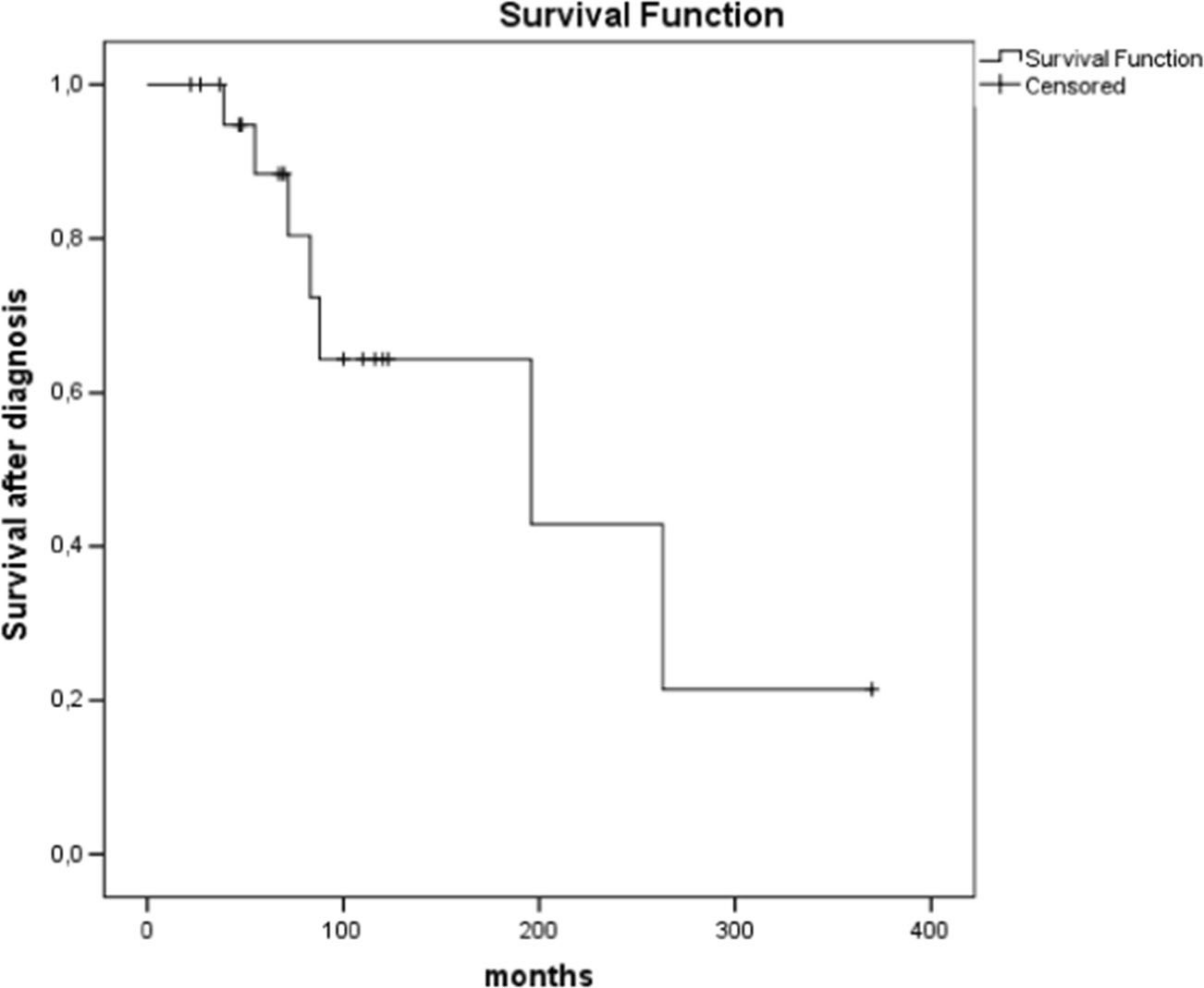
Kaplan-Meier analysis of survival after diagnosis

Twenty-one patients in this study were initially treated with microsurgical resection, while in two patients, transsphenoidal biopsy was performed as an initial diagnostic procedure, followed, however, by GTR in one patient. At the time of index surgery, the median tumour volume was 14 cm^3^ (range 0.9–75.0 cm^3^). We assessed the primary surgical outcome as GTR in 3 patients, STR in 12, partial in 6 and biopsy only in 1 case. Of those having the index surgery performed at our institution (*n* = 15), three improved neurologically after the surgery, with eight experiencing no change in their clinical status. Five of the operated patients experienced temporary deterioration in their neurological state, most often caused by cranial nerve palsies (4 out of 5 patients), which became permanent in two patients. Two experienced CSF leakage, of which one developed meningitis (complication grade II according to Ibanez). Two of the patients operated transsphenoidally developed pituitary deficiency (complication grade I according to Ibanez). Furthermore, one patient experienced an aggravation of previous respiratory failure necessitating intensive care (complication grade III according to Ibanez), while another experienced a postoperative haemorrhage requiring surgical intervention and intensive care treatment (complication grade III according to Ibanez). No peri-operative mortality, defined as death within 30 days after surgery, was seen in this series. Overall 5- and 10-year survival after diagnosis was 82% (14/17 patients) and 50% (6/12 patients), respectively. Three of nine (33%) patients survived more than 15 years. A Kaplan-Meier analysis revealed a median survival time of 196 months (CI 95% 3.5–388.5 months). Fifteen of 22 patients were alive at the time of follow-up, thus presenting an overall survival of 68%. Seven of 22 patients died during follow-up. Tumour progression was considered the cause of death in all but one patient who died from a radiation complication (patient no. 3). See Table 1 and Fig. 1 for more details.

### Gamma Knife treatment

**Fig. 2:**
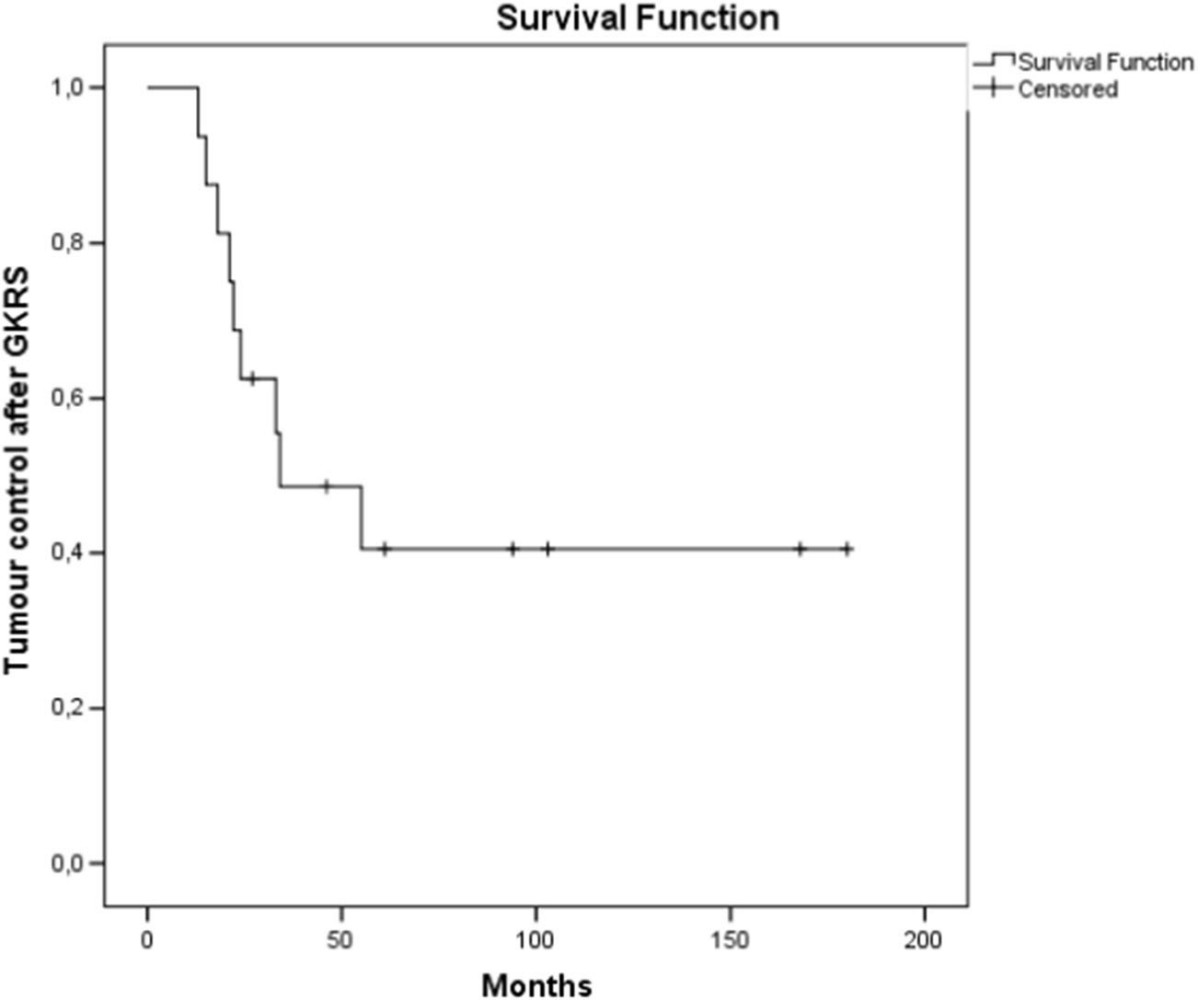
Kaplan-Meier analysis of tumour control after Gamma Knife radiosurgery

Of the 16 patients treated with GKRS, tumour control was achieved in 50% (8/16 patients) after the first GKRS treatment, with a median follow-up time of 34 months (range 6–180 months) after GKRS. The GKRS procedure was performed at a median of 22 (range 7–116) months after initial surgery. Kaplan-Meier analyses estimated a median tumour control of 34 months (95% CI 0–70 months). Loss of TC after GKRS was seen in 8/16 cases after the first GKRS (Fig. 2). After the first GKRS treatment, TC was achieved in 8/16 patients at 6 months (*n* = 1), 2–5 years (*n* = 3) and 8–15 years (*n* = 4), while recurrences were detected in 8/16. Four of these eight patients received a salvage microsurgical procedure at a median of 45 months after the initial GKRS, while the remaining four were treated with additional GKRS only. Of the recurrent tumours in eight patients, all grew outside of the initial GKRS prescription dose volume, while one recurrence was “in field”. An adverse radiation effect (ARE), seen as local edema or cranial nerve palsy, was seen in 3/16 patients treated with GKRS (Table 2). Six of the eight patients with failed radiosurgical control received additional GKRS. After the second GKRS, one patient had TC at 6-month follow-up, two had out-of-field recurrences at 21 and 2 months, respectively, and the remaining three patients had in-field tumour recurrence after 16–24 months.

**Table 2:** Gamma Knife radiosurgery treatment data

Patient no.	Year of GKRS	Indication for GKRS (tumour residual or tumour recurrence)	GKRS Tumour Volume cm^3^	Prescription dose (Gy)/ max dose (Gy)	Isodose	TC/ LTC (in or out of the radiation field)	Follow-up time (months)/ time to LTC (months)	ARE
1	2010	Residual	8.1	16/32	50	LTC/out of field	34	No
	2013	Recurrence	2.0	16/32	50	LTC/in field	19	No
2	2005	Residual	1.3	15/30	50	LTC/out of field	55	No
	2010	Recurrence	16.3	18/36	50	LTC/out of field	21	Yes
4	2010	Recurrence	3.7	18/36	50	TC	61	Yes
5	1990	Recurrence	6.0	20/40	50	TC	180	No
6	2008	Recurrence	4.7	19/38	50	TC	46	No
8	1992	Residual	6.0	12/30	40	TC	168	No
9	2002	Recurrence	2.1	20/40	50	TC	103	No
10	2005¤	Recurrence	2.2	14/28	50	LTC/ out of field	22	No
	2007¤	Residual	NA	ND	ND	LTC/ out of field	LF	No
	2008	Recurrence	11.4	16/36	45	LTC/out of field	LF	No
12	1993	Recurrence	NA	12/50	ND	LTC/out of field	33	Yes
	1996	Recurrence	24.3	15/38	40	LTC/in field	24	No
13	1996	Residual	5.1	14/35	40	LTC/out of field	21	No
14	1996	Recurrence	17.2	15/38	40	LTC/out of field	15	No
15	2006	Recurrence	2.0	16/32	50	TC	94	No
17	2010	Residual	9.2	16/29	55	TC	27	No
18	2015	Residual	4,0	18/36	50	TC	6	No
19	2013	Recurrence	16.0	18/36	50	LTC/out of field	24	No
	2015	Recurrence	4.0	18/36	50	TC	NA	No
21	2012	Recurrence	2,5	18/36	50	LTC/out of field	13	No
	2013	Recurrence	0,8	18/36	50	LTC/ in field	16	No

*Abbreviations*: *GKRS* Gamma Knife radiosurgery, *TC* tumour control, *LTC* loss of tumour control, *ARE* adverse radiation effect, *NA* not available, *LF* lost to follow-up

### Fractionated radiation and proton beam therapy

Seven of 22 patients received postoperative conventional FRT and/or proton beam radiotherapy (45–55 Gy). Proton beam radiotherapy was delivered as a salvage treatment following repeated surgeries during a course of 84 months in one (patient no. 2) and as adjuvant treatment after partial or STR removal in three patients (nos. 1, 3, 16) and after biopsy in one patient (no. 22). Patient no. 2 experienced tumour recurrence after 32 months, patient no. 1 after 51 months and patient no. 16 after 31 months. In patient no. 22, harbouring a chondroid chordoma (proliferation index 1%), TC was achieved at 123 months and TC was also achieved in patient no. 3, who died from radiation complications. Patient no. 3 developed an edema in the brainstem 18 months after the proton beam radiotherapy and deteriorated neurologically with hemi-paresis, confusion and blurred consciousness. The edema was considered an adverse radiation effect (RTOG grade 5) and the patient died 12 months later. Two patients received FRT as part of the initial treatment (no. 5) or as salvage therapy after recurrence (no. 13). Patient no. 5 had tumour recurrences treated with microsurgery and GKRS after 60 and 70 months, respectively, while patient no. 13 died from tumour progression 41 months after FRT. In patients treated with FRT or proton beam radiotherapy five of seven patients experienced a recurrence(s) necessitating salvage micro- and/or radiosurgery (Table 1).

#### Long-term tumour control

Our long-term figures allowed identification of six patients who did not show sign of residual disease, with tumour control >10 years (120–370 months) after treatment, which may contradict the belief that all chordomas recur if you allow for sufficiently long follow-up. Among the patients considered disease-free, three patients are still alive, two died from unrelated causes 120 and 293 months after treatment, respectively, while one was lost to follow-up after 10 years. Three of these patients underwent STR followed by GKRS, one underwent STR followed by FRT, another underwent GTR without any adjuvant treatment, and the last one with a histologically non-aggressive (chondroid chordoma, proliferation index 1%) small tumour underwent biopsy and proton-beam RT.

## Discussion

Our findings suggest (1) that adjuvant radiation therapy can be postponed or even replaced by GKRS for potential residuals or follow-up with serial imaging and GKRS treatment of recurrences, (2) radical surgery is not a prerequisite for long-term tumour control, (3) SRS can be used to control chordoma residuals or recurrences and to avoid repeated microsurgery, and (4) there is no obvious qualitative difference in biological response between photon- and proton-based RT.

### Timing of adjuvant RT and long-term survival

Our treatment results do not agree with the belief that early adjunctive radiotherapy is necessary in chordoma management. Our management comprised serial imaging follow-up with GKRS as needed upon recurrence or GKRS due to tumour residual after index microsurgery in 17/22 patients; it showed similar or even superior overall survival than reported in studies on maximal safe surgery followed by immediate adjuvant radiation therapy for clival chordomas. During follow-up, additional micro- or GKRS was used in case of tumour recurrence and/or progression. In the present study 5-year overall survival was 82%. This result is better than the mean overall 5-year survival of 54% for series with immediate adjuvant photon radiotherapy and similar to the 80–82% found in series implementing proton-beam, ion-particle or stereotactic radiation therapy [2]. Other researchers report worse long-term outcomes than Amichetti et al. Registry data from the UK [59] showed 56% 5-year survival and a recent systematic review showed an estimated 5- and 10-year survival of 63% and 16%, respectively [24]. Our 10-year survival of 50% compares equally with the best reported 10-year survival of 54% following proton beam [37] and 56% following surgery + GKRS. Furthermore, some reported series fail to differentiate between low-grade chondrosarcomas and chordomas [20] [37]. Mixed survival data will show falsely optimistic tumour control rates, since low-grade chondrosarcomas have a different and more benign course [1, 13, 15, 33]. Our data show that individual treatments were successful in a majority of patients, but also that we were unable to achieve long-term tumour control in a third of our patients.

### Surgical radicality and long-term tumour control

Although aiming for a safe maximal cytoreductive surgery for the initial treatment of patients with skull base chordoma, several reports on chordoma resection report a high number of surgical complications [1, 47, 56]. As chordomas progress, bony structures of the cranial base are destructed, the dura often becomes infiltrated and cranial nerves, and blood vessels will be enveloped. To achieve an STR or GTR of the chordoma in this situation, a combined or a staged resection is frequently needed [18, 34]. We used staged approaches in 7 of 12 patients with large tumours and 1 of 10 patients with a small tumour. We still considered risks associated with radical removal of infiltrated tumour margins as too high. Accordingly, these procedures could not be considered radical even with GTR achieved. We found many new neurological deficits (25%) after surgery, but only 12% became permanent. No deficit resulted in loss of independence (Karnoffsky <70). Less aggressive surgery correlates with low morbidity in cavernous sinus meningiomas [17]. We consider our results neurologically favourable, but only 3 patients underwent GTR, 12 had STR and 7 had 50% removal. Non-radical surgery is less aggressive, but usually increases risks of recurrence. Unexpectedly, our long-term figures allowed identification of six patients who we consider disease-free after >10-year follow-up. However, five out of six of these patients were treated with more aggressive surgery, indicating that maximal tumour removal is important for long-term tumour control.

The extradural and midline origin of chordomas makes a midline approach ideal for these tumours. In earlier reports, and also in the first cases of this series, an anterior transbasal approach was used [7, 11]. The development of endoscopic techniques during the last years has made the endonasal transsphenoidal approach the preferred choice for the index surgery [12, 14, 25, 28, 50]. It is technically easier to achieve extensive removal with other than transsphenoidal approaches, but our tumour control, when implementing transsphenoidal approach in combination with GKRS was comparable to when more extensive surgical approaches were used.Both strategies appeared to offer long-term tumour control and even cure in some patients. It is inevitable that microsurgical techniques will develop further and allow better microsurgical radicality without aggravating morbidity. Tamura et al. recently reported long-term results from extensive microsurgery followed by GKRS for recurrences [53]. They report 10- and 15-year survival rates of 72%, which is high compared to our results and the previous literature. Their surgical approach was more radical than ours, however, with notably higher complication rates. In accordance with our own strategy and results, Tamura et al. also followed their patients closely and re-treated when recurrences appeared. The management of these tumours as chronic diseases with close follow-up and readiness to re-treat is another important factor that seemingly improved long-term control and survival.

A final area of uncertainty is tumour biology. The biological behaviour varied between apparent cures in 25% of patients to death from intractable tumours in another 25%. This indicates a biological heterogeneity among chordomas. Proliferation indices varied widely in the few patients where it was analysed, and it also appeared that our two patients with chondroid chordomas did extraordinarily well. In agreement, particularly favourable outcomes for chondroid chordomas have been reported by others [6, 41]. The biological behaviour is thus not predictable from the diagnostic label “chordoma”. Proliferation indices, genotypic and phenotypic markers may allow prognostic sub-classification to determine therapeutic choices and clinical prognosis [22].

### Role of Gamma Knife radiosurgery and radiotherapy in chordoma treatment

In our cohort, radiation therapy was not given as an adjuvant therapy after first surgery. Instead small remnants of chordomas were treated with GKRS, as described in other chordoma series [22, 53]. FRT or proton beam radiotherapy was reserved as salvage treatment in case of tumour progression or recurrences after microsurgery and GKRS. With this treatment strategy, fractionated radiotherapy, which can usually only be administered once, could be saved until a later stage of the disease and was not necessary for most (15/22) of our patients.

A tumour control of 50% after the first GKRS, for a median follow-up time of 34 months, represents results in the lower end compared with previous published reports on GKRS use for clival chordoma residuals and recurrences [9, 19, 22, 26, 32, 33, 53, 61]. However, after GKRS, only 4 of 16 patients in this study needed new microsurgical treatment, which suggests a role for GKRS in the management of clival chordomas.

The literature contains a number of retrospective case series of microsurgery and adjuvant radiation. Our observations indicated that to achieve tumour control or cure, one does not necessarily need to implement immediate adjuvant photon or proton beam radiotherapy. The control rates for GKRS for chordoma residuals and tumour recurrences were similar or better in our series than in chordoma series with other treatment modalities, indicating that early radiosurgery of chordoma residuals did not provide any clear benefit compared to our management with “wait and scan” followed by tailored treatment of tumour recurrence if needed. In fact, three of four patients died from tumour recurrences or radiation necrosis within a few years after early proton beam treatment and half of the patients in the present series needed additional microsurgery and/or GKRS for recurrence within 5 years after early photon radiotherapy.

Our recurrence rate was 50% following initial radiosurgery in 16/22 patients. The recurrences occurred outside of the treatment field in eight of nine patients, which again reflected the infiltrative growth pattern of this locally aggressive tumour. Our observations following additional recurrences suggested gradual biological progression. Six of the eight patients received additional GKRS treatments, and this time three of six patients experienced tumour recurrence within the treatment field, without the possibility for further treatment, ultimately leading to death.

### Possible qualitative differences of adjuvant radiation

To avoid recurrences, FRT has been used although several reports indicate that conventional radiotherapy with a total dose <45–55 Gy is of limited value [8, 43]. Proton beam radiotherapy gives a steeper radiation gradient, which allows doses of 60–80-Gy equivalents to the targeted chordoma, with less risk of overdosing radiosensitive organs in the close vicinity, such as the brainstem and the optic nerves [40, 58]. Early results after proton beam radiotherapy are promising, but follow-up times are generally short in these series. Subsequently, late recurrences, which are the rule for chordomas, can go by undetected [48]. In a systematic review, Amichetti et al. [2] concluded that proton beam radiotherapy was superior to LINAC-based radiotherapy or radiosurgery for clival chordoma radiotherapy [4, 20, 21, 37, 40] [3, 39]. It is unclear whether the benefit is argued to reflect dose distribution or qualitative aspects of protons compared to photons, although the latter is frequently suggested. There were, however, methodological shortcomings in Amichetti’s systematic review: (1) no head-to-head comparative study on chordomas treated with LINAC-based RT or proton beam radiotherapy was included, (2) short follow-up times were reported in the included studies, and finally (3) higher radiation doses were usually used in the proton beam series (66–83 Gy). With modern LINAC techniques intensity-modulated arc therapy is combined with multileaf collimators. With these new technical developments, identical dose plans can usually be achieved with fractionated, LINAC-based RT as for dose plans for proton beam therapy. We therefore disagree with Amichetti’s conclusion that proton RT is superior to LINAC-based RT for clival chordomas. In agreement with our view, LINAC treatment with higher radiation doses (60–70 Gy) produced results comparable to the proton series [10]. Hadron therapy has been suggested to provide a qualitatively better effect, but so far long-term data are lacking and short-term data are similar to other modalities [48, 55]. Taken together, neither previous studies nor observations in our material show a qualitative difference in biological response between photons and protons. Instead, it appears that the dose distribution and ability to deliver sufficient radiation to the actual tumour, which is more readily done for small than large targets, determine the radiation response. In agreement, GKRS can be expected to be efficient for small circumscribed lesions because of the sharp dose gradient and feasibility of high radiation doses in the targeted tumour.

### Strengths and limitations of the study

The standard limitations of retrospective observational studies can also be identified in the present study. All retrospective heterogeneous series reflect uncoordinated individual treatment decisions determined by present states of knowledge and an ambition to provide best available care to individual patients. The lack of standardised treatment and small series make many statistical comparisons and evaluations invalid. It is, however, still possible to compare whether our results and observations agree with general treatment recommendations and beliefs—which also reflect an ambition to formulate the best possible options. In addition, robust and objective endpoints are relevant. Also, our study has a strength in renewed analyses of all tumour samples for confirmation of the diagnosis, which excluded two patients since pathological review and renewed staining led to a reclassification of two tumours as chondrosarcomas.

To avoid selection bias, all patients were included consecutively, without excluding patients with short follow-up. To avoid detection bias we used survival as the primary endpoint rather than progression-free survival or neurological deterioration, which are more subjective. Further, the long follow-up time in this study insured the detection of late events. Symptom development and side effects are more difficult variables and in this study we were dependent on documented events in the patients’ charts. Still, the patients had their follow-up at our institution, with the disease course documented in our own charts, which is better than in many retrospective multicentre studies. Unfortunately, median follow-up after GKRS was only 34 months in this series, and one has to suspect that later recurrences will emerge. A 3-year median follow-up after RT is, however, common in the literature on this topic. The present results on tumour control are comparable to RT reports with similar follow-up time [10, 40, 48]. The small number of study patients made regular statistical analysis impossible and results have instead been reported as the proportion of the total number of patients in the study.

## Conclusion

In the present consecutive series, we report on multidisciplinary management in a cohort of patients with clival chordomas, with emphasis on the use of GKRS to treat tumour residuals or as needed upon tumour recurrence. Overall survival for this cohort was 68% (15/22), with a Kaplan-Meier estimation of median survival at 196 months. Only 4/16 patients treated with Gamma Knife later needed re-operation and 6 patients were disease-free more than 10 years after the last treatment.

Our findings confirm previous reports in the literature indicating that clival chordomas should be viewed as a chronic progressive disease, with a continuous need to monitor and deal with potential recurrences, irrespective of adjuvant radiation. It is thus neither realistic nor rational to plan for a one-time cure comprising maximal safe surgery and adjuvant radiation [19, 22, 53]. Furthermore, our data suggest a beneficial role for Gamma Knife treatment in the multidisciplinary management of clival chordomas.
